# Bacteriology and Antibiotic Susceptibility Patterns among Neonates Diagnosed of Omphalitis at a Tertiary Special Care Baby Unit in Western Uganda

**DOI:** 10.1155/2020/4131098

**Published:** 2020-10-26

**Authors:** Munanura Turyasiima, Martin Nduwimana, Gloria Kiconco, Walufu Ivan Egesa, Silva Andres Manuel, Peter Kalubi, Yamile Enedina Arias Ortiz

**Affiliations:** ^1^Department of Pediatrics and Child Health, Faculty of Clinical Medicine and Dentistry, Kampala International University, Kampala, Uganda; ^2^Department of Pediatrics and Child Health, Faculty of Medicine, Mbarara University of Science and Technology, Mbarara, Uganda

## Abstract

**Background:**

Newborn infections remain a major cause of morbidity and mortality among neonates in low-income countries. Clinical diagnosis for omphalitis in such settings is possible but this does not depict the microbiological characteristics of the involved organisms, and clinicians have often prescribed empirical antibiotics in neonates with omphalitis, despite an increasing burden of antibiotic resistance.

**Methods:**

A hospital-based cross-sectional study was conducted to evaluate the bacteriology and antibiotic susceptibility patterns among neonates diagnosed with omphalitis at the special care baby unit (SCBU) of Kampala International University-Teaching Hospital (KIU-TH), western Uganda from March to June 2019. Sixty-five (65) neonates with a clinical diagnosis of omphalitis were consecutively recruited in the study. Cord swabs were taken under sterile (aseptic) precautions from all neonates, and antibiotic susceptibility tests performed using the Kirby Bauer disk diffusion technique with commercially available antibiotics disks of ampicillin, cloxacillin, gentamicin, amikacin, cefotaxime, ceftriaxone, vancomycin, and imipenem on Mueller Hinton agar plates. The data was analyzed using STATA version 13.0, frequencies and proportions used to describe the variables.

**Results:**

Fifty-five, 55 (84.6%), neonates with suspected omphalitis had positive cord swab culture. Staphylococcal aureus (58.2%) was the commonest cause of omphalitis followed by Neisseria spp (16.4%), E. coli 6 (10.9%), Proteus *spp* (5.5%), Klebsiella *spp* (3.6%), Citrobacter *spp* (3.6%), and Haemophilus *spp* (1.8%) in decreasing frequency. Isolates were resistant to ampicillin (87.7%), gentamicin (54.4%), and cloxacillin (34.4%), the drugs recommended for use in neonates with suspected omphalitis.

**Conclusions:**

Staphylococcal aureus is still the predominant cause of omphalitis among neonates. There was high resistance to the commonly used antibiotics in the treatment of omphalitis among newborns. This study reemphasizes that clinicians should do cord swabbing for both culture and susceptibility tests among newborns with suspected omphalitis before initiation of antibiotics.

## 1. Background

Neonatal omphalitis is still a common condition in low resource settings where routine care of the umbilical stump still involves application of potentially dangerous substances [1–3], contrary to the cord care recommendations by the World Health Organization [4]. The high mortality rate from omphalitis, due partially to the rapid progression from omphalitis to necrotizing fasciitis, can be prevented through early recognition of the signs and symptoms of omphalitis followed by prompt, aggressive treatment [5].

Diagnosis of omphalitis through physical examination of the umbilicus is an acceptable standard of diagnosis according to WHO [4]. This, however, does not describe the causative organisms and hence precise antibiotic treatment. The result is that many clinicians now use empirical antibiotics for every neonate whose focus for sepsis is infection of the umbilicus, and this has contributed to the growing global antibiotic resistance. Since neonates are vulnerable to drug toxicity, and few specific drugs are recommended for use in this group, it is important that culture and sensitivity patterns of the causative organisms involved in omphalitis be optimized while treating neonates.

While Staphylococcus *aureus* remains the most frequent bacterial cause of omphalitis in neonates, other many bacteria have been isolated from cord swabs cultures of neonates with omphalitis including *Escherichia coli*, *Klebsiella* species, *Pseudomonas* species, group A and group B *Streptococci*, and Tetanus [6–13]. Without proper and specific identification of the causative organism and treatment, through culture and antibiotic susceptibility tests, the risk of antibiotic resistance will continue to increase.

A systematic review by Huynh and colleagues [14] on community-acquired invasive bacterial infections and antibiotic resistance among neonates in developing countries found available data insufficient to draw a true, recent, and accurate picture of antibiotic resistance among severe bacterial infections in neonates, particularly at the community level. This article adds to the few epidemiological reports on the bacteriology and antibiotic susceptibility patterns of the commonly used antibiotics among neonates diagnosed with omphalitis in low resource settings.

## 2. Methods and Materials

### 2.1. Study Design and Setting

This was a hospital-based cross-sectional study on neonates diagnosed with omphalitis at a special care baby unit (SCBU) of Kampala International University-Teaching Hospital (KIU-TH), Uganda. KIU-TH is a tertiary teaching and referral hospital in western Uganda, located approximately 326.6 km southwest of Kampala, the city of Uganda. Sixty-five (65) neonates with a clinical diagnosis of omphalitis were consecutively recruited in the study from March to June 2019. The study evaluated the bacteriology from cord swab cultures and antibiotic susceptibility patterns among neonates daiganosed of omphalitis.. This study did not evaluate for sepsis that may result from omphalitis, and so only cord swabs but not blood samples were taken for culture from the neonates.

### 2.2. Inclusion and Exclusion Criteria

The study was conducted in term and late preterm neonates. Term neonates already started on antibiotic therapy at the time of recruitment were excluded.

### 2.3. Definition of Neonatal Omphalitis

Omphalitis was diagnosed in every neonate found to have at least one physical sign of purulent discharge, reddening, and swelling (edema) on the physical examination of the cord stump according to WHO neonatal guidelines [4].

### 2.4. Collection of Samples

Cord swabs were taken under sterile (aseptic) precautions from all neonates with clinical evidence of omphalitis, before administering antibiotic therapy. Cord swab tip was rotated over 1 cm^2^ area of the deep part of septic cord stump for 5 seconds (Levine method) while avoiding touching the stump edge with the swab. Infection control during swabbing was observed through hand washing, use of sterile gloves, cleansing the surrounding (periumbilical area), and use of sterile swab sticks and gloves for each client. The samples were delivered to the KIU-TH laboratory on the same day of sample collection to minimize contamination.

### 2.5. Culture of Samples

*Smear preparation*: inoculation was first made on the culture media before using the swab to make smears for Gram staining.

*Gram staining*: the specimen was evenly spread on a clean, grease-free slide and allowed to air-dry in a safe place. The specimen was heat fixed and stained by *Gram staining technique*. The smear was then examined for the presence of bacteria and pus cells (PMNs) using 100x objective lens and specially looking for Gram-negative rods; Gram-negative diplococci; Gram-positive cocci in pairs, chains, or clusters; and Gram-positive large rods with square ends.

### 2.6. Susceptibility Tests of Isolated Specimens

The antimicrobial susceptibility tests were performed using the Kirby Bauer disk diffusion technique (Bauer et al., 1966) with commercially available disks on Mueller Hinton agar plates.

The following antibiotic disks were used: ampicillin (10 *μ*g), cloxacillin (5 *μ*g), gentamicin (10 *μ*g), amikacin (30 *μ*g), cefotaxime (30 *μ*g), ceftriaxone (30 *μ*g), vancomycin (30 *μ*g), and imipenem (10 *μ*g). The diameter of the zone of inhibition for each antibiotic was measured and interpreted as resistant, intermediate, and sensitive according to Clinical Laboratory Standards Institute Criteria (2007).

### 2.7. Data Interpretation and Analysis

The data was analyzed using STATA version 13.0, frequencies and proportions used to describe the variables under study.

## 3. Results

### 3.1. Neonates' Perinatal Characteristics

Sixty-five (65) neonates met the WHO diagnosis criteria of omphalitis. Most of the neonates were male, aged below 7 days of life with a normal average birth weight, and not exposed to maternal perinatal infection. This is shown in Table 1.

### 3.2. Bacterial Isolates from Cord Swabs of Neonates Diagnosed with Omphalitis

**Figure 1:**
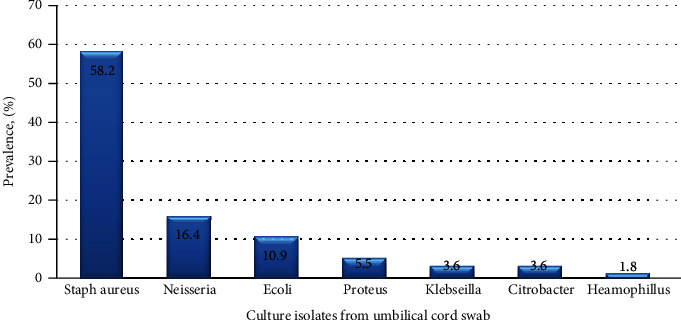
Bar graph showing the percentage prevalence of bacteria causing omphalitis among neonates.

Fifty-five, 55 (84.6%), of the 65 neonates with clinical signs of omphalitis had positive culture results. Staphylococcus aureus 32(58.2%), the only gram-positive isolate, was the most frequent isolated organism. The rest of the isolates were Gram-negative bacteria (41.8%) and included Neisseria *spp*9 (16.4%), E. coli 6 (10.9%), Proteus *spp*3 (5.5%), Klebsiella *spp*2 (3.6%), Citrobacter *spp*2(3.6%), and Haemophilus *spp*1 (1.8%). This is summarized in Figure 1.

### 3.3. Percentage Antibiotic Susceptibility Patterns of Bacterial Isolates from Cord Swabs of Neonates Diagnosed of Omphalitis

The susceptibility of isolated organisms to commonly used antibiotics including ampicillin, cloxacillin, gentamicin, amikacin, cefotaxime, ceftriaxone, vancomycin, and imipenem was assessed and reported as sensitive, intermediate, and resistant.

**Figure 2:**
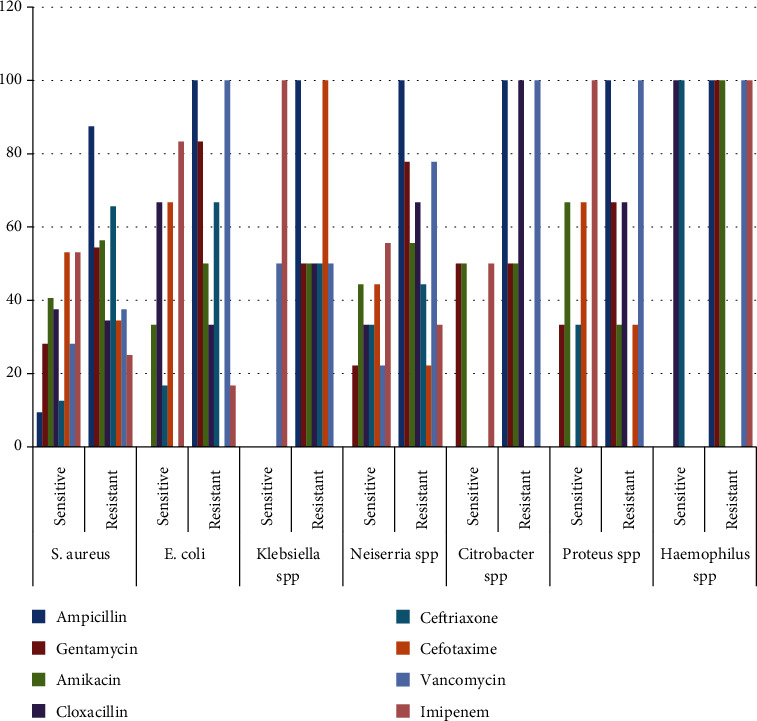
Percentage antibiotic sensitivity and resistance patterns of bacterial isolates from cord swabs among neonates diagnosed of omphalitis.

The findings generally show a high antibiotic resistance across all the isolated organisms. The highest antibiotic sensitivity of the isolated organisms is as follows; Staphylococcal *aureus*, the most frequently isolated organism, was only moderately sensitive (53.1%) to cefotaxime and imipenem; Neisseria *spp* moderately sensitive (55.6%) to imipenem, low sensitivity (44.4%) to amikacin and cefotaxime; E. *coli* high sensitivity (83.3%) to imipenem and moderate sensitivity (66.7%) to cloxacillin and cefotaxime; Klebseilla *spp* highly sensitive (100%) to imipenem and moderate sensitivity to vancomycin (50%); Proteus *spp* had moderate sensitivity (66.7%) to amikacin and cefotaxime; Citrobacter *spp* only had moderate sensitivity (50%) to amikacin and gentamicin. Haemophilus spp were highly sensitive (100%) to cloxacillin, and ceftriaxone, yet highly resistant (100%) to ampicillin, gentamicin, amikacin, imipenem, and vancomycin. This is shown in Table 2 and Figure 2.

## 4. Discussion

Empirical use of ampicillin/gentamicin and cloxacillin/gentamicin combinations is recommended for presumed sepsis and sepsis where staphylococcal infection is highly suspected (for example in omphalitis), respectively, whereas blood and/or swab cultures should be collected before antibiotic administration where possible [4]. Staphylococcal *aureus* was the most frequent bacterial isolate of all the cord swabs of neonates with omphalitis in this study. This finding is universal to all other epidemiological studies on omphalitis etiology and neonatal related sepsis worldwide [6, 14]. The high frequency of staphylococcal aureus isolates (58.2%) in this study is comparable to other studies, 39.8% in Uganda [8] and 57.9% in Pakistan [9]. Staphylococcal aureus showed moderate sensitivity to cefotaxime and imipenem (53.1%), and these could be used instead of the commonly used resistant ampicillin (87.5%), gentamicin (54.4%), and cloxacillin (34.4%) that showed high resistance. Staphylococcus *aureus* is a normal flora in the mothers' birth canal; nonsterile hands of the persons involved in maternal delivery [6] and hence can easily contaminate the umbilicus of the newborn during delivery. In addition, most cord care practices in low resource settings involve the application of potentially dangerous substances to the umbilicus of the neonates [1–3], which results in the high rate of staphylococcal *aureus* transmission from these nonsterile substances.

Gram-negative bacteria are on average the commonest etiology for omphalitis than Gram-positive [14], unlike the findings in this study where Gram-positive bacteria were predominant. A related study among neonates with cord sepsis at Mulago national referral hospital (Uganda) [8] found Gram-negative bacteria the most predominant isolates (60.4%) mainly due to *E. coli* (33.3%), *Proteus* (14.6%), and *Klebiesella* (10.4%). A prospective study in India also found Gram-negative organisms to be responsible for the majority of omphalitis in 57.1%, of cases, and Klebsiella spp were the most isolated [12]. Neisseria *spp* (16.4%) was the second frequent isolate and the most common Gram-negative bacteria in this study. This finding is peculiar as few or no study has isolated Neiserria spp from umbilical cord swabs. Either E. *coli* or Klebsiella *spp* is the second most isolated organism after Staphylococcal aureus from cord swabs of neonates with omphalitis in all previous studies under literature search [6–13]. This finding could suggest a correspondingly high incidence of gonococcal infection among mothers of the neonates involved in this study. Other organisms like Proteus *spp* (5.5%), Klebsiella *spp* (3.6%), Citrobacter *spp* (3.6%), and Haemophilus *spp* (1.8%) were isolated from cord swabs of neonates in this study at lower frequencies. Although Klebsiella *spp* were isolated in a few swabs in this study, it caused most of the omphalitis among neonates in a study done in India [12] in which Gram-negative organisms were responsible for the majority of the omphalitis.

In this study, all the bacterial isolates from the cord swabs generally showed a high antibiotic resistance and low sensitivity pattern to the commonly used antibiotics in omphalitis. Staphylococcal aureus, the most common isolate, was highly resistant to ampicillin, gentamicin, and cloxacillin at 87.5%, 54.4%, and 34.4%, respectively. There was, however, a low resistance to cefotaxime and imipenem, 34.4% and 25%, respectively, and these two drugs could be used to treat staphylococcal omphalitis instead of cloxacillin and gentamicin. The 2015 situation analysis on antibiotic resistance in Uganda [15] estimated staphylococcal *aureus* resistance to ampicillin between 40 and 100% and resistance to gentamicin of up to 30%. A hospital study in Tanzania also found a high resistance to ampicillin and cloxacillin, moderate resistance to ceftriaxone and cefuroxime, and low resistance to amikacin among bacterial isolates from cord swabs of neonates [7]. Neiserria *spp*, the second common isolate in this study, showed a high resistance to the commonly used ampicillin (100%), gentamicin (77.8%), and cloxacillin (66.7%). There was similarly high antibiotic resistance patterns among all the other bacterial isolates from the cord swabs.

The high rates of resistance in this study are in line with the national antibiotic resistance situation (over 50 percent in many cases) by a broad range of bacteria to commonly used antibiotics such as penicillin, tetracycline, and cotrimoxazole [15]. A high prevalence of antimicrobial self-medication frequently associated with inappropriate drug use in developing countries [16], including Uganda, contributes to this high antibiotic resistance. Routine culture and sensitivity prior to administration of empiric antibiotics could be a solution; however, it remains a dream in resource-limited settings. In such circumstances, clinicians blindly prescribe cloxacillin/gentamicin combination to treat omphalitis where staphylococcal infection is highly suspected and ampicillin/gentamicin when it is unlikely. For settings where cord swabbing for culture is possible, culture/sensitivity reports come late because conventional methods are commonly used. This still makes it impossible for the clinicians to follow the right treatments at the right time of prescription.

This study did not describe resistance by methicillin-resistant Staphylococcus aureus (MRSA) and extended-spectrum beta-lactamase (ESBL)-producing bacteria that confer resistance to some advanced antibiotics. A high-level study is recommended to discuss this public health problem.

Primary prevention of omphalitis through clean delivery, clean cord care, avoiding harmful cord care practices and by increasing tetanus toxoid immunization coverage can potentially reduce the prescription of antibiotics among neonates. This in turn could reduce the bacterial resistance to the commonly used antibiotics in neonatal omphalitis. World Health Organization [4] recommends daily chlorhexidine (4%) application to the umbilical stump during the first week of life for newborns born at home in settings with high neonatal mortality (neonatal mortality rate >30 per 1000 live births) and clean dry cord care for newborns born in health facilities, and at home in low neonatal mortality as the primary prevention of omphalitis.

## 5. Conclusions

Staphylococcus *aureus* was the predominant cause of omphalitis among neonates admitted in the special care baby unit. Neisseria *spp* was the second frequent isolate and the common Gram-negative cause of omphalitis, indicating a possibly high rate of gonococcal infections among the nursing mothers in this setting. There was high antibiotic resistance to the commonly used antibiotics in neonates with omphalitis. Cord swabs for culture and sensitivity should always be taken in all neonates with signs of cord infection before antibiotics administration to minimize irrational antibiotic use and resistance. This study did not evaluate sepsis and related mortality that may result from omphalitis. More studies are required to further describe the resistance patterns in many settings and as well influence the current treatment policies.

## Figures and Tables

**Table 1:** Perinatal characteristics of neonates diagnosed of omphalitis.

Characteristic	Frequency, *n* (%)
Age of the neonate	
<7 days	60 (92.3)
7-14 days	5 (0.7)
Neonatal birth weight (g)	
≤2499	5 (7.7)
2500-3999	50 (76.9)
≥4000	10 (15.4)
Sex of the neonate	
Female	36. (55.4)
Male	29 (44.6)
Mode of delivery	
Vaginal	23 (35.4)
Caesarean	42 (64.6)
Gestational age (weeks of amenorrhea)	
Term (>37)	60 (92.3)
Preterm (≤36)	5 (0.7)
Place of delivery	
Home	2 (3.1)
Private health facility	55 (84.6)
Public health facility	8 (12.3)

**Table 2:** Percentage antibiotic susceptibility patterns of bacterial isolates from cord swabs of neonates diagnosed of omphalitis.

	Ampicillin	Gentamicin	Amikacin	Cloxacillin	Ceftriaxone	Cefotaxime	Vancomycin	Imipenem
S. aureus								
Sensitive	9.4	28.1	40.6	37.5	12.5	53.1	28.1	53.1
Intermediate	3.1	17.5	3.1	28.1	21.9	12.5	34.4	21.9
Resistant	87.5	54.4	56.3	34.4	65.6	34.4	37.5	25.0
E. coli								
Sensitive	0	0	33.3	66.7	16.7	66.7	0	83.3
Intermediate	0	16.7	16.7	0	16.6	33.3	0	0
Resistant	100	83.3	50.0	33.3	66.7	00	100	16.7
Klebsiella spp								
Sensitive	0	0	0	0	0	0	50.0	100
Intermediate	0	50.0	50.0	50.0	50.0	0	0	0
Resistant	100	50.0	50.0	50.0	50.0	100	50.0	0
Neiserria spp								
Sensitive	0	22.2	44.4	33.3	33.3	44.4	22.2	55.6
Intermediate	0	0	0	0	22.3	33.4	0	11.1
Resistant	100	77.8	55.6	66.7	44.4	22.2	77.8	33.3
Citrobacter spp								
Sensitive	0	50.0	50.0	0	0	0	0	50.0
Intermediate	0	0	0	0	100	100	0	50
Resistant	100	50.0	50.0	100	0	0	100	00
Proteus spp								
Sensitive	0	33.3	66.7	0	33.3	66.7	0	100
Intermediate	0	0	0	33.3	66.7	0	0	0
Resistant	100	66.7	33.3	66.7	0	33.3	100	0
Haemophilus spp								
Sensitive	0	0	0	100	100	0	0	0
Intermediate	0	0	0	0	0	100	0	0
Resistant	100	100	100	0	0	0	100	100

## Data Availability

The datasets used to support the findings of this study are available from the corresponding author upon request.
